# Should GDMT be prioritized over revascularization in new onset HFrEF? Potential lessons from the REVIVED-BCIS2 and STRONG-HF trials

**DOI:** 10.3389/fcvm.2023.1193226

**Published:** 2023-06-12

**Authors:** Neal M. Dixit, Ezra A. Amsterdam

**Affiliations:** Division of Cardiovascular Medicine, School of Medicine, University of California, Davis, Sacramento, CA, United States

**Keywords:** guideline-directed medical therapy, heart failure, revascularization, cardiomyopathy, coronary artery bypass surgery, percutaneous coronary intervention

## 1. Introduction

Many patients with heart failure with reduced ejection fraction (HFrEF) are initially diagnosed at an index heart failure hospitalization. Nearly half of these patients have an ischemic etiology. In the latter group, the Surgical Treatment for Ischemic Heart Failure (STICH) trial was the first, and thus far, the only trial to show benefit of revascularization on survival in patients with left ventricular (LV) dysfunction (1). Revascularization by percutaneous coronary intervention (PCI) has also been applied to this population in anticipation of achieving similar benefit. The REVIVED-BCIS2 trial tested this hypothesis by randomizing patients with multivessel coronary artery disease (CAD) and a left ventricular ejection fraction (LVEF) < 35% to PCI and guideline directed medical therapy (GDMT) vs. GDMT alone (2). However, no benefit on survival or LV function was found with PCI compared to GDMT during a mean follow-up interval of more than 3 years. Thus, for patients with HFrEF and stable multivessel CAD, coronary artery bypass graft surgery (CABG) is the only revascularization method with documented mortality benefit. GDMT remains the foundation of care for these patients.

This finding raises an important question: For newly diagnosed HFrEF patients at high risk for CAD not presenting with acute coronary syndrome, how should revascularization be prioritized at index hospitalization?

## 2. Discussion

### 2.1. GDMT should be the focus for newly diagnosed HFrEF

The 2022 ACC/AHA/HFSA Guidelines identify 4 pillars of GDMT for HFrEF (3). Each of these agents has shown reduction in heart failure hospitalization and cardiovascular death within 30 days of initiation (4). However, due to widespread underuse of GDMT, there have been considerable and potentially avoidable losses of life and function (5). The Guidelines suggest simultaneous initiation of this regimen at diagnosis with subsequent titration at regular intervals (3). This is a documented strategy to reduce both early and long-term mortality and morbidity in HFrEF, regardless of etiology.

In fact, the STRONG-HF trial randomized patients hospitalized with heart failure, a majority with HFrEF, to a strategy of aggressive initiation and titration of GDMT vs. usual care (6). The result was a notable 8.1% absolute risk reduction in the primary endpoint of 180-day readmission for heart failure or all-cause death. N-terminal pro-brain natriuretic peptide was also reduced by 23% at 90 days in the aggressively titrated arm compared to usual care despite no difference in doses of loop diuretics. The key to this success was targeted up-titration of GDMT peri-discharge, resulting in >80% of patients on half-target dose or greater of beta-blocker, renin-angiotensin-aldosterone system inhibitor, and mineralocorticoid receptor antagonist by 2 weeks post-discharge.

While an ischemic evaluation is a crucial aspect in the evaluation of newly diagnosed HFrEF, immediate revascularization may impede GDMT initiation. Percutaneous coronary intervention**-**induced acute kidney injury (AKI) occurs in up to 7%–10% of cases, which may prohibit initiation or continuation of GDMT agents, which often transiently reduce glomerular filtration rate (7). Moreover, the concern for contrast induced AKI from coronary angiography can render clinicians reluctant to titrate GDMT. Additionally, GDMT use after CABG has historically been lower than with PCI, presenting another barrier to medical optimization (8).

### 2.2. Could there be benefit to deferred revascularization of multivessel disease?

Guidelines provide a Class I indication for revascularization with CABG for patients with high risk left main (LM) CAD and multivessel CAD associated with diabetes or LVEF < 35% (9). However, as with candidacy for implantable cardiac defibrillators for primary prevention, consideration of revascularization may shift as a patient's LVEF improves after optimization of GDMT (10). Per Guidelines, a patient with an LVEF of 30% with multivessel disease and no diabetes has a Class I indication for CABG; however, in three months if the EF improves to 35%–50% with optimal GDMT, then the recommendation for CABG drops to Class 2a; if EF improves to >50% then it becomes 2b. Moreover, marked LV dysfunction is a leading reason for rejection of surgery due to the increased risk of surgical mortality (1, 5). Historically, many of these turndowns are sent for PCI. However, results of REVIVED-BCIS2 reveal that this approach may not have been beneficial (2). But even with high rates of surgical mortality, the STICH trial showed that the clinical benefits of CABG in LV dysfunction are eventually realized (1). Prioritization of optimal GDMT before revascularization in patients whose sole indication is LVEF < 35%, could result in increased LVEF at the time of consideration for CABG, which may obviate the need for CABG**,** or lower operative risk if the decision is made to proceed with CABG. Therefore, deferred, i.e., postponement of this decision to the outpatient setting, may be a preferred strategy for management of these patients.

### 2.3. Consider a non-invasive ischemic evaluation

**Figure 1:**
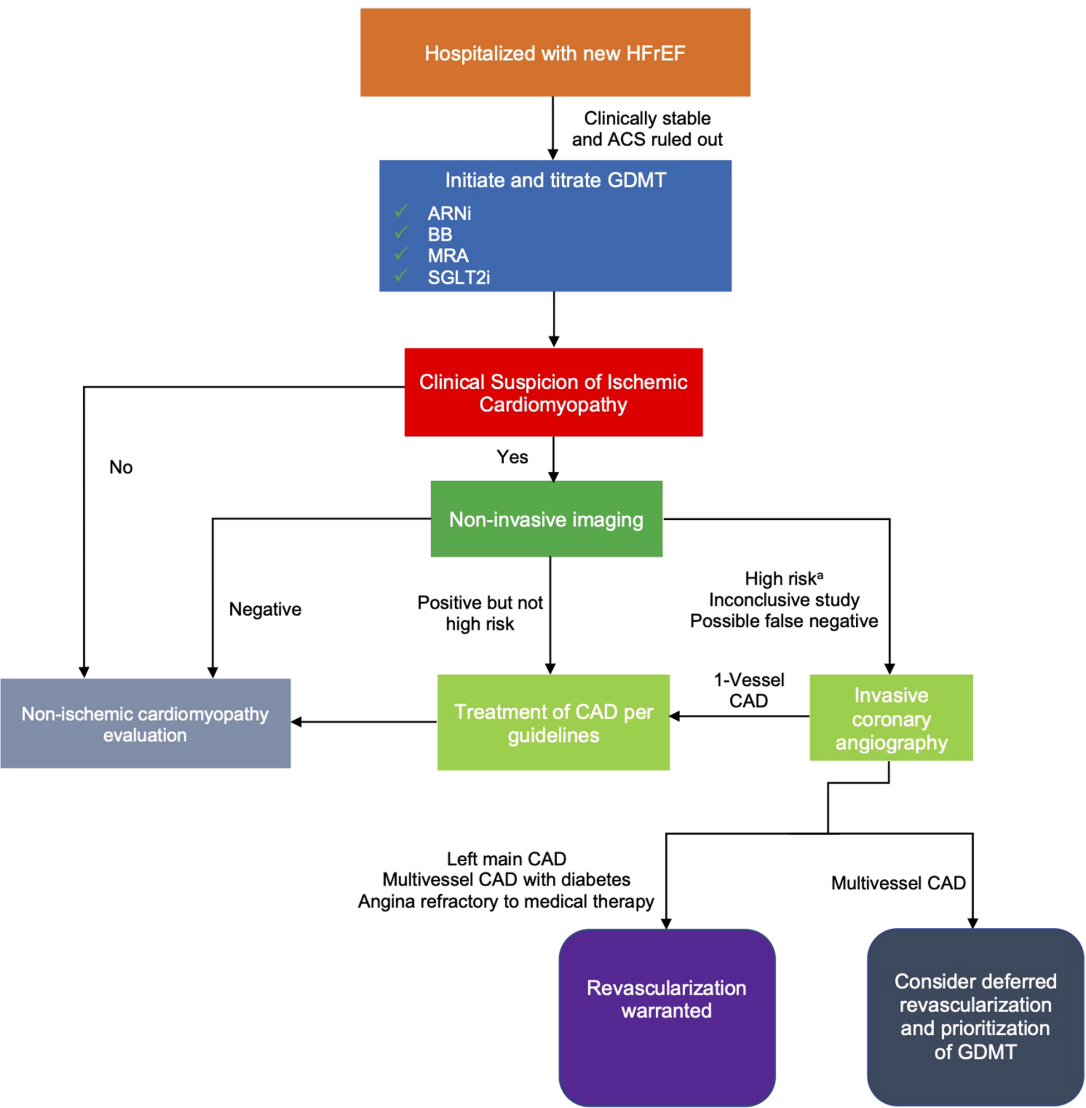
Proposed algorithm for evaluation of ischemic cardiomyopathy in hospitalized patients with newly diagnosed HFrEF. ^a^Suggestive of LM or multivessel CAD. ACS, acute coronary syndrome; ARNi, angiotensin receptor-neprilysin inhibitor; BB, beta-blocker; CAD, coronary artery disease; GDMT, guideline-directed medical therapy; HFrEF, heart failure with reduced ejection fraction; MRA, mineralocorticoid receptor antagonist; SGLT2i, sodium/glucose cotransporter-2 inhibitors.

While invasive coronary angiography has been the gold-standard for diagnosis of ischemic cardiomyopathy, the limited role of PCI in patients with LV dysfunction and stable CAD may limit its necessity. Non-invasive imaging minimizes procedural risk while maintaining diagnostic accuracy for high risk disease. Coronary computed tomography angiography (CCTA) has upwards of 90% sensitivity and specificity for identifying obstructive CAD (11). For certain patients in which CCTA may be impractical, such as those with elevated heart rates or marginal kidney function, non-invasive stress imaging can be used to detect LM and triple vessel CAD (3, 12). Patients with high risk, inconclusive, or high likelihood of false negative findings on non-invasive testing can be considered for invasive angiography, but the possible benefits of deferred revascularization should be considered as previously noted (Figure 1). Patients whose non-invasive testing is negative or not high risk have low annual rates of ischemic events and further invasive evaluation can be performed in the outpatient setting, if indicated (13, 14). In these cases, non-ischemic causes of cardiomyopathy should also be evaluated (15).

## 3. Conclusion

With the negative results of the REVIVED-BCIS2 trial, CABG remains the only method of revascularization in patients with ischemic cardiomyopathy to demonstrate morbidity and mortality benefit. However, deferred revascularization of multi-vessel disease in patients with new onset HFrEF should be considered to allow time for the impact of the rapid, beneficial effects of GDMT, which may lead to lower surgical risk or render CABG unnecessary. Additionally, an initial non-invasive ischemic evaluation reduces procedural risk and may better facilitate GDMT optimization than an initial invasive evaluation. Overall, for newly diagnosed HFrEF patients, a strategy prioritizing GDMT over revascularization may lead to greater long-term benefits. A randomized trial is required to provide further guidance on this approach.
